# PERCEIVED BALANCE PREDICTS FALLS IN COMMUNITY-DWELLING OLDER ADULTS

**DOI:** 10.1093/geroni/igae098.2720

**Published:** 2024-12-31

**Authors:** Hanne Dolan, Janet Pohl, Keenan Pituch, David Coon

**Affiliations:** Arizona State University, Phoenix, Arizona, United States; Arizona State University, Phoenix, Arizona, United States; Arizona State University, Phoenix, Arizona, United States; Arizona State Unviersity, Phoenix, Arizona, United States

## Abstract

Accidental falls are an increasing problem among older adults in the United States (US). Older adults’ self-report of falls is the primary method of fall risk identification. However, up to 72% of Medicare beneficiaries do not report falls and fall-related injuries to their healthcare providers. Limited research suggest that older adults prefer the term “balance problems” instead of “fall risk.” The aim of this study was to examine if perceived balance problem is a predictor of self-reported falls after controlling for known predictors of falls among older adults. The Health Belief Model and the concept of perceived susceptibility served as the theoretical framework. A longitudinal secondary analysis was conducted using data from a subsample of independently living participants (N = 5446) from the National Health and Aging Trends Study. Baseline data was from year 2015, and the outcome was self-reported falls in 2016, with 23.4% of the sample reporting a fall. Complex samples multiple logistic regression analyses revealed that perceived balance (OR = 1.69, p <.001) predicted falls in 2016, whereas, the balance performance measure, Short Physical Performance Battery, did not (OR = 0.98, p =.06). Also, non-Hispanic White participants were more likely to report falling compared to non-Hispanic Black and Hispanic participants, as were females compared to males. A stay in the hospital in 2015, co-morbidities, fear of falling, and reporting a fall in 2015 were also predictive of falls. Assessing older adults’ perceptions regarding their own balance is important to identify fall risk.

